# Experiences with pain of early medical abortion: qualitative results from Nepal, South Africa, and Vietnam

**DOI:** 10.1186/s12905-019-0816-0

**Published:** 2019-10-15

**Authors:** Daniel Grossman, Sarah Raifman, Tshegofatso Bessenaar, Lan Dung Duong, Anand Tamang, Monica V. Dragoman

**Affiliations:** 1 Advancing New Standards in Reproductive Health (ANSIRH), Bixby Center for Global Reproductive Health, Department of Obstetrics, Gynecology and Reproductive Sciences, University of California, San Francisco, USA; 2 Ibis Reproductive Health, Oakland, CA USA; 3 Ibis Reproductive Health, Johannesburg, South Africa; 4 Job Shimankana Tabane Provincial Hospital, Tlhabane, Rustenburg, South Africa; 5 National Hospital of Obstetrics and Gynecology (NHOG), Hanoi, Vietnam; 6 Center for Environment Health and Population Activities (CREHPA), Kathmandu, Nepal; 7 Paropkar Maternity and Women’s Hospital, Kathmandu, Nepal; 8 Department of Reproductive Health and Research, WHO, UNFP/UNDP/UNICEF/WHO/World Bank Special Programme of Research, Development and Research Training in Human Reproduction (HRP), Geneva, Switzerland; 9 Gynuity Health Projects, New York, NY USA

**Keywords:** Medical abortion, Pain, Nepal, South Africa, Vietnam

## Abstract

**Background:**

Medical abortion (MA) has become an increasingly popular choice for women even where surgical abortion services are available. Pain is often cited by women as one of the worst aspects of the MA experience, yet we know little about women’s experience with pain management during the process, particularly in low resource settings. The aim of this study is to better understand women’s experiences of pain with MA and strategies for improving quality of care.

**Methods:**

This qualitative study was conducted as part of a three-arm randomized, controlled trial in Nepal, Vietnam, and South Africa to investigate the effect of prophylactic pain management on pain during MA through 63 days’ gestation. We purposively sampled seven parous and seven nulliparous women with a range of reported maximum pain levels from each country, totaling 42 participants. Thematic content analysis focused on MA pain experiences and management of pain compared to menstruation, labor, and previous abortions.

**Results:**

MA is relatively less painful compared to giving birth and relatively more painful than menstruation, based on four factors: pain intensity, duration, associated symptoms and side effects, and response to pain medications. We identified four types of pain trajectories: minimal overall pain, brief intense pain, intermittent pain, and constant pain. Compared to previous abortion experiences, MA pain was less extreme (but sometimes longer in duration), more private, and less frightening. There were no distinct trends in pain trajectories by treatment group, parity, or country. Methods of coping with pain in MA and menstruation are similar in each respective country context, and use of analgesics was relatively uncommon. The majority of respondents reported that counseling about pain management before the abortion and support during the abortion process helped ease their pain and emotional stress.

**Conclusions:**

Pain management during MA is increasingly essential to ensuring quality abortion care in light of the growing proportion of abortions completed with medication around the world. Incorporating a discussion about pain expectations and pain management strategies into pre-MA counseling and providing access to information and support during the MA process could improve the quality of care and experiences of MA patients.

**Trial registration:**

Australian New Zealand Clinical Trials Registry ACTRN12613000017729, registered January 8, 2013.

## Background

Medical abortion has become an increasingly popular choice for women even where surgical abortion services are available. In areas where providers lack necessary skills or equipment needed for surgical abortion, medical abortion may enable access to safe abortion services. The most effective regimen for medical abortion (MA) is mifepristone followed 24 to 48 h later by misoprostol and can be provided at a primary care facility by non-physician providers [1]. Women prefer MA for reasons related to privacy, autonomy, and feeling that it is less invasive than surgery [2–6].

Pain is often cited by women as one of the worst aspects of the MA experience [5, 7], in addition to duration of bleeding, the number of visits required, and the waiting time to know if the treatment has been successful. Inadequate pain management may motivate some women to seek unnecessary clinical care. Pain perception is one of several patient factors beyond procedural safety that is significantly associated with patient experience and quality of care [8]. There is therefore a clear need to identify the most effective methods of pain control for MA patients, both for the purpose of improving abortion quality of care and reducing the financial and human resource burdens of unnecessary care-seeking. Improved quality of care will help ongoing efforts aimed at increasing availability and acceptability of MA, particularly in resource-poor settings.

Despite its importance to the abortion experience, little is known about optimal pain management during MA, particularly in low-resource settings [9–14]. Individual experiences and responses to pain and pain medication are complex and may differ according to ethnicity, socio-economic status, cultural factors, physiology and genetics. Further, there is variation in the pain management protocols followed by abortion providers around the world. The World Health Organization (WHO) and the Royal College of Obstetrics and Gynaecology (RCOG) currently recommend the use of non-steroidal inflammatory drugs (NSAIDs) during MA with mifepristone and misoprostol [15, 16]. Previous studies have shown that NSAIDs such as ibuprofen reduce pain once uterine cramping has started and are superior to paracetamol; however, ibuprofen does not appear to be effective at preventing or minimizing moderate to severe MA pain when administered alone prophylactically [17].

Further research is needed to determine best pain management options, including timing of medication administration, for MA provided before and after 12 weeks’ gestation [1]. In addition, there is a lack of evidence on MA pain management in diverse populations of women outside the United States, Canada and Israel [17, 18], where women may have very different experiences. This study aims to better understand women’s experiences of pain with MA in Nepal, South Africa, and Vietnam and the potential differences in pain experiences given variation in pain management strategy, parity, and country context. The results of this study may be used to improve quality of care in MA service provision and may be useful in other low-resource settings that are beginning to expand MA services.

## Methods

This qualitative analysis was conducted as part of a multi-center, three-arm, blinded, randomized, controlled trial to investigate the effect of prophylactic pain management on maximal pain in the first 8 h following misoprostol administration during MA through 63 days’ gestation. The trial was conducted in Kathmandu, Nepal; Rustenburg, South Africa; and Hanoi, Vietnam. Participants were randomized in a 1:1:1 allocation ratio to either one of two active prophylactic pain management arms (group 1: tramadol 50 mg and a placebo, or group 2: ibuprofen 400 mg and metoclopramide 10 mg) or a placebo arm (group 3) [19]. Study treatment was given at the time of misoprostol administration and repeated 4 h later. All participants were given 4 tablets ibuprofen 400 mg and 2 tablets paracetamol with codeine to take as needed for pain not treated by the study medication. In addition to collecting quantitative clinical and symptom data, we conducted in-depth interviews with a subset of participants to better understand perceptions of pain and effects of pain management.

Between June 2016 and October 2017, women were recruited in person at sites where safe and legal MA services were well-established: the Paropkar Maternity and Women’s Hospital in Kathmandu, Nepal; Job Shimankana Tabane Hospital in Rustenburg, South Africa; and the National Hospital of Obstetrics and Gynecology in Hanoi, Vietnam. Eligible participants included women 18 years and older seeking MA up to 63 days’ gestation, who were willing to participate in the study and were fluent in the language of the site, possessed sufficient literacy to adhere to study instructions and complete home self-assessments, and agreed to be contacted by telephone. Women with pregnancy abnormalities or allergies/contraindications to any of the study medications or who reported no access to a time-keeping device or telephone were excluded. Written informed consent was obtained from all participants before conducting the interviews. The full study protocol has been previously published [19].

We performed in-depth interviews with 7 nulliparous and 7 parous participants in each country. In order to capture varied pain experiences in the interviews for this qualitative analysis, and because young age and parity have been associated with pain levels during MA [20], we purposively sampled parous and nulliparous women with a range of reported maximum pain levels in the first 8 h after taking misoprostol as measured on an 11-point numeric visual analogue scale with values ranging from 0 (no pain) to 10 (worst possible pain). At each site, we calculated the median maximum pain level and aimed to recruit approximately three participants above that level and three below that level for each of the nulliparous and parous groups.

Participants were invited to participate in an interview at the follow-up visit at the facility approximately 2 weeks after initiating the MA. When possible, the participant completed the interview that same day; those who could not stay after their follow-up visit were scheduled for another time at the facility, between 5 days and 4 weeks after taking mifepristone. No repeat interviews were conducted. Interviews were conducted by TB, DLD, AT and four research staff (see Acknowledgements) in a private location and in the participants’ preferred language. Interviewers asked open-ended questions about pregnancy history and experience with general pain, menstrual pain, and pain in labor, methods for managing each type of pain, and specific pain experiences throughout the most recent MA process (interview guide available as Additional file 1). Interviews ranged in duration from 30 min to 1 h. The research team pilot-tested the interview guide in Nepal and made adjustments to the guide. Because these changes were minimal, these initial interviews were included in the analysis.

Interviewers were trained in qualitative research methods and ethics, and all were female (except DLD who completed 1 interview). Most interviews were conducted by staff who had a Bachelor’s or Master’s degree and were not involved in the clinical care of participants; two interviews were conducted by physicians in Vietnam, of which one was involved in the care of the participant. Interviewers informed participants of the goals of the research prior to obtaining written informed consent for conducting and audio recording the interview; they also informed participants that they were researchers not involved in their clinical care (except for the one case where the physician interviewer was involved). If participants declined to be recorded, the interviewer took notes instead. The interviewer also recorded or wrote field notes at the end of the interview. All participants received cash reimbursement for their travel to participate in the study interview, the amount of which varied by site.

Professionals transcribed and translated the interviews; transcripts were not returned to participants to review. The principal investigators reviewed each of their respective site’s transcripts for quality control purposes and provided feedback to the interviewers as needed. Two researchers developed a codebook primarily based on the interview guide, and one applied codes to all of the interviews using Dedoose Version 7.0.23 (SocioCultural Research Consultants, LLC, Los Angeles, CA); coding was not reviewed with participants. Researchers clarified queries in the transcribed data with the respective interviewer who completed the interview and referred to the interviewer’s additional notes taken at the time of the interview. The researchers coded for patient characteristics, pain tolerance, pain management (for general, menstrual, birth, and abortion pain respectively), pain levels and duration (for menstruation, birth, abortion), other side effects of medical abortion, reasons for abortion, support from others during the abortion decision-making process and the abortion itself, general reflections about the abortion, self-stigma around abortion as related to pain, and preferences for type of abortion (medical versus surgical).

In addition to describing participants’ physical experience with pain and the role of pharmacologic analgesics, we aimed to explore how psychological and social factors related to the abortion might influence this experience. Our approach is informed by the biopsychosocial model [21], which states that the pain experience may be influenced by biological, psychological, and social factors [22]. Themes were derived from the data using thematic content analysis, which included identifying themes, generating frequency counts of these themes, and comparing trends in these themes to the established literature. Though recruitment numbers were not determined by interview saturation, analysis revealed that saturation on key themes related to MA pain experiences and management of pain was reached. In the results, the following participant information is included in parentheticals: age category in years (y), country, parity (parous or nulliparous), and treatment group (tramadol, ibu/met, placebo).

## Results

The study team invited and interviewed a total of 42 participants, including 7 parous and 7 nulliparous women from each of the three study countries; no participant declined to be interviewed. All interviews were recorded except for one, where the recording device failed and the interviewer took notes instead. Participants included 15 from the tramadol group (3 in Nepal, 8 in South Africa, and 4 in Vietnam), 16 from the ibuprofen/metoclopramide (ibu/met) group (7 in Nepal, 3 in South Africa, 6 in Vietnam), and 11 from the placebo group (4 in Nepal, 3 in South Africa, 4 in Vietnam). The median age of participants was 23 (range: 18–44, IQR = 20-32). Most participants in South Africa were single (*n* = 13), while most participants in Nepal and Vietnam were married (Nepal: 11, Vietnam: 8) or partnered (Vietnam: 6). One-third of the sample had completed secondary school, and 64% had completed more than secondary school; 43% of participants were currently in school. All except 3 participants in South Africa lived in urban areas (see Table 1). Food insecurity was reported by only one woman in Nepal, who responded that she often went without food in her household.

**Table 1:** Characteristics of study participants

	Nepal (*n* = 14)	South Africa (*n* = 14)	Vietnam (*n* = 14)	Total (*n* = 42)
Age (median)	23.5	22.5	25.5	23
Marital status				
Single	2 (14%)	13 (93%)	0	15 (36%)
Married	11 (79%)	0	8 (57%)	19 (45%)
Partnered	0	0	6 (43%)	6 (14%)
Divorced/Separated	1 (7%)	1 (7%)	0	2 (5%)
Highest level education				
Primary	0	0	1 (7%)	1 (2%)
Secondary	5 (36%)	8 (57%)	1 (7%)	14 (33%)
More than secondary	9 (64%)	6 (43%)	12 (86%)	27 (64%)
Currently in school				
Yes	5 (36%)	7 (50%)	6 (43%)	18 (43%)
Primary occupation				
Student	2 (14%)	7 (50%)	6 (43%)	15 (36%)
Housewife	7 (50%)	1 (7%)	0	8 (19%)
Professional	5 (36%)	0	6 (43%)	12 (29%)
Unemployed	0	4 (29%)	0	4 (10%)
Other	0	2 (14%)	2 (14%)	4 (10%)
Residence				
Urban	14 (100%)	11 (79%)	14 (100%)	39 (93%)
Rural	0	3 (21%)	0	3 (7%)
Parity				
Nulliparous	7 (50%)	7 (50%)	7 (50%)	21 (50%)
Parous	7 (50%)	7 (50%)	7 (50%)	21 (50%)
Number previous pregnancies				
0	7 (50%)	7 (50%)	7 (50%)	21 (50%)
1	2 (14%)	3 (21%)	1 (7%)	6 (14%)
2	3 (21%)	3 (21%)	1 (7%)	7 (17%)
3 or more	2 (14%)	1 (7%)	5 (36%)	8 (19%)
Reported previous abortions (among those previously pregnant)				
0	3 (43%)	7 (100%)	1 (14%)	11 (52%)
1	4 (57%)	0 (0%)	6 (86%)	10 (48%)
Number of participants who previously had a medical abortion (among those previously pregnant)	2 (29%)	0	1 (14%)	3 (14%)
Number of participants who previously had a surgical abortion (among those previously pregnant)	2 (29%)	0	5 (71%)	7 (33%)
Treatment group				
Group 1 (tramadol 50 mg, placebo)	3 (21%)	8 (57%)	4 (29%)	15 (36%)
Group 2 (ibuprofen 400 mg, metoclopramide 10 mg)	7 (50%)	3 (21%)	6 (43%)	16 (38%)
Group 3 (2 placebo tablets)	4 (29%)	3 (21%)	4 (29%)	11 (26%)

Among parous participants, the average number of previous pregnancies was 2 in Nepal, 1.7 in South Africa, and 3.1 in Vietnam. One-quarter of the sample reported having a previous abortion (only in Nepal and Vietnam), and three participants had previous MA experience. The average gestational age at presentation was 49 days (SD: 7.8, range: 34–63 days); Nepal and Vietnam had similar averages (47 and 46 days respectively) while South Africa’s average was 55 days. On a 10-point scale, the average overall pain level reported with MA was 5.2 (SD: 2.5). The average highest pain in the first 8 h after misoprostol was 6.6 (SD: 2.5), ranging from 6.1 in Nepal to 6.7 in Vietnam and 7.1 in South Africa. All participants had a successful medical abortion without any further treatment.

The following four themes emerged from the content analysis: pain and other side effects of the MA; medical abortion pain relative to menstrual, labor, and previous abortion pain; pain management; and emotional experiences (see Table 2). We explore these four themes below.

**Table 2:** Themes identified in content analysis

Theme	Sub-themes/details
Pain and other side effects	Pain trajectory • Minimal pain • Brief intense pain • Intermittent pain • Constant pain Other side effects: chills/shivering, nausea/vomiting, fever, diarrhea
Medical abortion pain relative to menstrual, labor, and previous abortion pain	Pain characteristics • Intensity • Duration • Associated symptoms and side effects • Response to pain medications Most participants considered pain with medical abortion worse than menstrual pain. Most participants who experienced childbirth considered labor pain worse than medical abortion Supportive providers may lessen pain experience.
Pain management	South African participants reported general use of paracetamol and ibuprofen for pain, while those in Nepal and Vietnam less commonly used medications. Non-medicinal pain management included wrapping a piece of cloth around one’s abdomen, eating or drinking hot foods and liquids, and using a hot water bottle or massage.
Emotional experiences	Range of emotions described: minimal emotional response, relief, guilt, ambivalence. Most participants thought emotional response did not worsen physical pain experience. Emotional support from family and friends improved abortion experience, including physical pain. Those who had previous abortions felt psychologically more prepared for pain of medical abortion.

### Pain and other side effects

Based on participant descriptions, we identified four types of pain trajectories. Ten respondents reported *minimal pain* overall (4 Nepal, 4 South Africa, 2 Vietnam). One said, “I never had an abortion, so I was expecting more pain but there was no pain. It was just normal; I was doing the house chores I am used to doing” (31–35 y, South Africa, parous, placebo). Another explained, “It wasn’t painful at all, not even a little. There was no such feeling of pain. Only when the pregnancy started to discharge, there was a feeling of something coming out, like when I’m on my period … but there was absolutely no pain” (31–35 y, Vietnam, parous, tramadol).

Eight participants reported *brief intense pain* and said it occurred right before expulsion. One participant said, “It hurt terribly, it hurt in a way that it’s very unlike any normal pain … I had 30 minutes of intense pain, after that I felt better, then gradually it eased and then I could walk as usual” (31–35 y, Vietnam, parous, tramadol). Nine participants experienced *intermittent pain*, which some described as similar to labor contractions: “It pained and disappeared and again pained and again disappeared” (21–25 y, Nepal, nulliparous, placebo). Another participant explained, “I think it’s like labor pain, intermittent pain from light pain to heavy pain” (31–35 y, Vietnam, parous, ibu/met). And five participants described feeling *constant pain* for an hour up to several hours: “The pains were like 10 … for about 3 or 4 hours … there was no change. The pains were constant” (18–20 y, South Africa, nulliparous, ibu/met). There were no distinct trends in pain trajectories by treatment group, age, parity, or country.

The most commonly reported symptoms were chills and shivering (12), nausea (9), vomiting (8), fever (8), and diarrhea (8), with no clear trends by parity, treatment group, or country. One participant said, “I felt really cold, and I was shivering even when I was staying in the sun, it still felt cold” (18–20 y, Nepal, nulliparous, ibu/met). Another reported, “Diarrhea was the most intolerable. It made my stomach gurgle and I felt nauseous so I needed to go to the bathroom constantly even though I felt cold and just wanted to stay in my bed … the diarrhea and nausea were the worst” (21–25 y, Vietnam, nulliparous, tramadol). Five participants reported weakness and/or dizziness: “I tolerated the pain for half an hour, it was all because of dizziness that I couldn’t tolerate the pain” (21–25 y, Nepal, nulliparous, tramadol). Five participants said they had numbness or immobility (under the tongue or in the limbs). One woman described, “…it felt so numb with pain…I think it was because of the pills melting. I think they made me feel that way” (36–40 y, South Africa, parous, tramadol). Another said, “…There was no abdominal pain. Only my limbs, it felt like I can’t handle it anymore, I felt paralyzed…It hurt terribly, it hurt in a way that it’s very unlike any normal pain, I have never experienced such kind of pain. My limbs couldn’t even move” (31–35 y, Vietnam, parous, tramadol). All but one participant in Vietnam and over half of the Nepal participants (9) reported at least one symptom beyond pain, while only 6 participants in South Africa reported symptoms.

### Medical abortion pain relative to menstrual, labor, and previous abortion pain

MA was reportedly more painful than menstruation for most participants who commented on the comparison regardless of parity or treatment group (23 vs. 9 who reported MA as less or similarly painful compared to menstruation) and less painful than labor (17 vs. 4 who reported MA as more or similarly painful compared to labor). Respondents compared overall pain of the current MA with pain in menstruation, labor, and previous abortion using one or more of the following factors: pain intensity, pain duration, associated symptoms and side effects, and response to pain medications. One woman ranked her experiences in terms of intensity and duration: “… the least painful was suction abortion, at level 7-8 but only for a short period of time. The second one would be the recent [medical] abortion, heavy bleeding hurt at level 9-10. Third one is my second labor, pain level was at 7-8 but lasted longer than the abortion. And my first labor hurt the most, pain level 9-10 and lasted incredibly long” (31–35 y, Vietnam, parous, ibu/met).

With regard to pain intensity, one participant said, “This one wasn’t that bad at all, I didn’t even feel like I was doing an abortion, there wasn’t a lot of pains. So the menstruation one is the worse one if I were to compare them” (21–25 y, South Africa, parous, ibu/met). In contrast, another said, “the abortion gave me heavy pain…it was more painful than the period pains” (36–40 y, South Africa, parous, tramadol). Several participants who had previous abortions attributed lower pain levels in the current abortion to the use of pain medications: “The bleeding was same on both times. But the pain was more in previous abortion than this time. I also didn’t have much difficulty as I took [pain] medicine this time…Maybe that is why compared to the previous abortion I didn’t feel much pain” (26–30 y, Nepal, parous, tramadol). Another said that just having pain medications easily available to her made the experience better: “During my previous abortion, I wasn’t told to do anything if I had pain. So this time I had medicine if I had pain, even though I didn’t take it… so it felt really good. Instead of going out and buying the medicine, when one has a packet with themselves they can easily take it” (36–40 y, Nepal, parous, ibu/met). One more explained, “If I have to compare, [this time] was painful for less time while using medicine … during my last abortion…it was 6-7 on scale for 6-7 days. But this time even if the pain was 10 on scale, it got lessened after having medicine” (31–35 y, Nepal, parous, ibu/met).

Most parous participants explained that the pain intensity of labor was much higher than MA: “It was nothing compared to my labor pain, because my labor pain was extreme…the labor is the worst, then comes the suction abortion, finally the pain from this abortion using medicine” (31–35 y, Vietnam, parous, ibu/met). Another explained, “labor pains were higher…maybe 5 to 1. Labor pain 5 and abortion pain 1” (21–25 y, South Africa, parous, tramadol).

Duration of pain in menstruation was shorter and typically predictable for most participants, whereas with MA, it was less predictable and occurred throughout the experience. One participant explained, “For menstruation, it pains a little on the first day and then it doesn’t pain. But during abortion, it was paining on the first day and on the third day” (26–30 y, Nepal, parous, tramadol). Another said, “I don’t get pain during every menstruation…I feel pain for one day only. During abortion as well, I had pain for a day only. That is why I feel it’s the same” (31–35 y, Nepal, parous, ibu/met). Those who reported previous abortions often determined which experience was more painful by comparing duration of bleeding and pain. One participant said she preferred her previous MA to the current one because the time it took to expel the pregnancy was longer this time and her pain was delayed (41–45 y, Vietnam, parous, placebo). Similarly in childbirth, participants attributed higher overall pain to a longer duration of pain: “when I delivered my child, the whole day I felt like the time when I had my abortion” (31–35 y, Nepal, parous, ibu/met).

For many, the comparison of pain in MA with that of menstruation or labor was dependent on symptoms other than pain alone. For example, one participant said, “My period pain is just normal, but [with MA] it was twisting and I had diarrhea and fever also, chills and shaking. That’s the difference” (21–25 y, Vietnam, nulliparous, tramadol). Another said, “Normal delivery is painful because of many reasons, for example episiotomy….because there’s no factors like that, MA is much less painful” (41–45 y, Vietnam, parous, placebo). Another added, “With giving birth, beside labor pain, you suffer also from the tear of vagina afterward, which prolonged the pain. After the abortion, you only suffer for 1 day” (26–30 y, Vietnam, parous, ibu/met).

Ten participants (6 Vietnam, 4 Nepal) reported a previous abortion and compared it to their current experience. Some women explained that the lack of instruments helped reduce their pain or anxiety levels with the current MA compared to a previous surgical abortion. One woman said, “…this time hurt more than last time but was less scary because I didn’t have to listen to the sound of the surgical tools, so there was less mental pain… the clanking sound of surgical tools scared me” (31–35 y, Vietnam, parous, ibu/met). Another said it “was much less painful, my psyche was much at ease, and it was more private…I feel quite embarrassed every time I have gynecology check. Like taking my clothes off and checking, that’s what makes me feel not comfortable at all” (31–35 y, Vietnam, parous, ibu/met). Another who had experienced three surgical abortions highlighted the importance of supportive, non-judgmental providers:

“*In my other previous abortions there was just me in the room. The doctors were scary and the things they said really annoyed me, like ‘… I don't want to do this. It's just that you asked for it,’ … so I felt very uncomfortable. …I had to accept it. I had a wonderful consultation this time, I was able to do self-check at home, and I had my relatives next to me and also someone to talk to during the process. Time went by really fast. I felt stronger mentally not feeling like abandoned in the room like any of those previous abortions…this time was more painful but more relieved. I felt safer since the surgical ones are scary, not as painful but much more scary.*” (31-35 y, Vietnam, parous, tramadol).

One participant said the current abortion was worse than her previous experience because she had an incomplete abortion that required her to have multiple follow-up visits (although she did not have additional treatment).

### Pain management

Most reported that the study medications eased MA pain. One participant said, “Because they not only gave me medicine when I was in pain but before I had pain they had given me medicine. So I felt my pain got cured after taking medicine. I didn’t have to take additional medicine” (21–25 y, Nepal, parous, tramadol). However, it was not always clear if participants were referring to the study medications or the additional analgesics taken as needed as being responsible for the pain relief. One respondent said, “It was very satisfying because if it wasn’t for the medication I would have slept in pain” (21–25 y, South Africa, nulliparous, tramadol). Another commented, “After having the medicine, it lessened my pain and I felt like eating as well. I had a fear that if something might happen, after the pain was gone, I felt that I am fine now” (31–35 y, Nepal, parous, ibu/met).

For managing pain in general (not specific to MA), some South African women reported taking medications including paracetamol and aspirin; only a few women in Nepal and Vietnam reported taking medications for more extreme pain. A woman from Nepal said, “If sometimes I have more pain and I have to go for duty, I take paracetamol. Otherwise I don’t...If it is paining around 5, I take medicine” (36–40 y, Nepal, parous, ibu/met).

The use of non-medicinal methods for managing MA pain were most common in Nepal, and less so in South Africa and Vietnam. These methods were similar to those reported for menstrual pain, including most commonly wrapping a piece of cloth around one’s abdomen (Nepal), eating or drinking hot foods and liquids (all countries), and using a hot water bottle or massage (all countries). One woman said, “I was dependent on hot water…my husband used to give me water frequently. He boiled the water in a thermos and put a glass in front of me. I just took rest by drinking hot water” (41–45 y, Nepal, parous, ibu/met). Participants in Nepal and Vietnam also reported eating warm foods with protein, such as soup or eggs, to ease pain. One woman in Vietnam explained, “Only my sister massaged me. She massaged and pressed on the area where I said was painful” (31–35 y, Vietnam, parous, tramadol). In South Africa, several women reported taking hot baths and lying flat on the floor to soothe the pain: “I just lay down on a cold floor and they become better” (31–35 y, South Africa, parous, tramadol), and “I just took a bath to relax my body” (31–35 y, South Africa, parous, tramadol).

One participant explained the use of a cloth wrap around the abdomen for menstrual pain:

…*Because there is just air in our stomach, [so] we have pain in stomach. That is why it gets better after wrapping a piece of cloth. ... I wrap it for 1-2 hours when I have pain. …. When I wrap it like that it lessens my back pain as well. When I have menstrual period, we have stomach and back pain, so it also lessens that pain. I haven’t used any [medicine] to date*. (21–25 y, Nepal, parous, tramadol)

In South Africa, a participant said, “I take the hot water bottle and I put it there or something that is warm and eventually I become okay…it helps and sometimes it does not…I sleep if nothing works” (21–25 y, South Africa, nulliparous, ibu/met). One respondent in Vietnam said she tried taking a menstrual regulation pill, a combination of herbal extracts containing traditional medications called Phu Huyet Khang in between periods to prevent pain: “The next period, the pain was much less intense… this medicine helps regulate the menstrual cycle” (21–25 y, Vietnam, nulliparous, placebo).

For managing labor pain, many reported walking, keeping busy, and massaging their bodies during labor; only three reported receiving pain medications for labor pain (one in South Africa and two in Vietnam). One woman said, “…my mother massaged me with oil all over my stomach…my mother told me that the pain might lessen if I stayed in a hanging position…but I just stayed clenching my teeth” (26–30 y, Nepal, parous, tramadol). Another woman answered, “Nothing, I was just walking and screaming” (31–35 y, South Africa, parous, placebo). A participant in South Africa said, “With my first child I asked them to bring me black forest [cake]…I would eat, when that pain comes…I ate that thing almost twelve hours… with my second-born I was better…I realized that I should nurse the pain. The more you walk the more you relieve stress” (36–40 y, South Africa, parous, tramadol).

### Emotional experiences

Many participants said they felt conflicted or guilty about having an abortion, but no one expressed regret about the decision. One woman said, “Half of me wanted to have an abortion and the other half wanted to keep the child, but I couldn’t…I stayed strong and went forward because I could not just sit and let the pain take over, I had to get up and get going, as sitting and crying will not help” (31–35 y, South Africa, nulliparous, placebo). Another participant said, “And after I had the abortion, I felt happy and secured…I didn’t feel anything emotionally. I didn’t want this pregnancy, I only thought about how this pregnancy will go and I will feel relieved” (31–35 y, Nepal, parous, ibu/met). In Vietnam, one woman said, “I feel like I’m such a terrible human being” for having an abortion (18–20 y, Vietnam, nulliparous, ibu/met), and another explained, “I feel guilty about what I have done, yet I think it was the best thing that we could do provided our situation” (21–25 y, Vietnam, nulliparous, placebo). Others were not emotional at all about the experience: “I didn’t have any feelings as such. I just wanted it to finish as quickly as possible” (31–35 y, Nepal, parous, ibu/met). Another explained, “If I had thought of keeping it then I would have been emotional. But when I had thought of having abortion, then I didn’t feel anything about it” (Nepal, parous, tramadol, 21–25y). One woman said, “I just wanted to get rid of this pregnancy. That’s all I was thinking” (21–25 y, Nepal, nulliparous, placebo).

Most who struggled emotionally or were conflicted said their emotions did not affect their physical pain. However, many explained that having emotional support from family and friends during the process made them feel more secure and in some cases more physically at ease. One woman from Nepal said, “If our husband is with us, then it will be a lot easier” (21–25 y, Nepal, nulliparous, tramadol). Another Nepali woman said, “Because I was feeling really bad from inside, so my emotional pain was a lot more than my physical pain, so I think that was the reason that helped me to not focus on my physical pain” (21–25 y, Nepal, nulliparous, ibu/met). Several participants from Vietnam discussed the importance of family or partner support:

*I think the mental state really affects how we feel during abortion. If we’re more relaxed, then we will feel less pain. Maybe I was unhappy and not so comfortable about this, so I felt more pain. … if [patients] have someone to comfort them that would be the best mental remedy to reduce the pain* (21-25 y, Vietnam, nulliparous, placebo).

*I felt that I wasn’t abandoned, so that pain was better...I can feel less scared even when my boyfriend just holds my hands* (18-20 y, Vietnam, nulliparous, placebo).

*Basically when you're taking that pill you'd better have someone next to you. That's for the best…. Firstly, just in case something happens there would be someone next to you to calm you down and help you. Secondly, it's better to have someone to talk to rather than being alone* (31-35 y, Vietnam parous, tramadol).

In South Africa, participants shared similar sentiments. One participant said, “I cried a bit in the afternoon and at night I was fine because my friend was there” (18–20 y, South Africa, nulliparous, placebo), and another said, “I don’t think I would have coped [without mama]. Imagine if I had to go get the water on my own, get the pain killers, the water bottle, all of that” (18–20 y, South Africa, nulliparous, ibu/met).

Those who lacked support explained that it would have helped. One participant said, “I contained everything within myself and I did not tell anyone, so I am thinking maybe if I had shared with someone I do not think I would have felt that much pain” (21–25 y, South Africa, nulliparous, ibu/met). Another explained:

*I was the only one who knew…There was no one to help me, no one to talk to….I wish we had already been married so he could be by my side and everyone could know. If the pain got worse, everyone could have taken care of me or told me what to do.* (21–25 y, Vietnam, nulliparous, tramadol)

Women who had previous experience with abortion appeared more prepared psychologically to handle the MA pain. For example, one woman said, “I accept that pain because I know it should happen like that. It is what I must accept because I had reckoned it before….because I was prepared beforehand, I already knew all the steps, the pain that I had to endure, I can mentally be ready to overcome it easily…so I can be less nervous” (31–35 y, Vietnam, parous, tramadol). Another said, “I had to abort it anyhow so my focus was on abortion only. I felt normal as I had done abortion previous to this as well” (36–40 y, Nepal, parous, ibu/met).

## Discussion

Participants in this multi-country study reported that MA is relatively less painful compared to giving birth and relatively more painful than menstruation, based on four factors: pain intensity, duration, associated symptoms and side effects, and response to pain medications. Compared to previous abortion experiences, MA pain was less intense (but sometimes longer in duration), more private, and less frightening. The lack of notable differences identified in pain experiences by treatment group, country and parity are likely due to our small sample size and the fact that participants were purposively sampled with a range of pain scores; analysis of quantitative data indicates that there are significant differences in pain by treatment group (manuscript under review).

Methods of coping with pain in MA and menstruation are similar in each respective country context. Medications are generally not the first-line strategy for pain relief for MA, menstruation or labor. In labor, alternative methods of pain management appear less common than in menstruation or MA; the focus is on tolerating pain rather than on pain management. In these countries, it is possible that use of pain medications is related to limited knowledge of analgesic options, restricted availability, high cost, provider unwillingness to prescribe medications for these indications, and lack of cultural acceptance of pain medication [23–25].

At the time of abortion, a variety of psychosocial and emotional factors such as anxiety, depression, and anticipation of pain influence women’s experiences with pain [26–28]. One study found that pre-abortion depression and younger age were significant predictors of pain intensity in surgical abortion [26]. Participants in the present study did not attribute pain levels directly to their emotional state before or during the abortion process; however, the majority reported that support from family and friends was helpful in easing pain and emotional stress.

Nonpharmacological interventions, such as “vocal local,” supportive verbal communication and distraction through conversation, can be helpful during first-trimester surgical abortion [29]. In a randomized controlled trial of 101 women undergoing first-trimester surgical abortion, participants were randomized to music through headphones or to usual care; though pain scores were similar between groups, two-thirds of the music group thought the intervention reduced their pain and anxiety and 93% would choose to listen to music again [30]. Doula support during abortion care may also address patient psychosocial needs during abortion. A randomized controlled trial found that doula support during first-trimester surgical abortion did not have a measureable effect on pain or satisfaction, but women receiving doula support were less likely to require additional clinic support resources and almost all (96%) recommended it for routine care [31, 32]. Though these interventions have not been applied specifically to MA, it is reasonable to expect they may reduce pain and anxiety in MA as well. Although potentially helpful for some patients, doula support is not necessary for the safe provision of MA and may not be feasible in all settings.

Comprehensive standardized counseling on pain in MA may also be an effective intervention in managing pain during the process. Little is known about what counseling or information is provided, particularly for prophylactic analgesics, before MA. An international survey of MA providers (primarily based in Europe) found that most do provide analgesia routinely to women undergoing MA up to 9 weeks gestation, either prophylactically (82%) or upon request (12%), and that analgesics (NSAIDs, paracetamol) are most often used in both cases [33]. Six percent of providers indicated that they never provide analgesics (or prescriptions for them), and female compared to male providers of abortion care were significantly more likely to prescribe routine analgesia for patients (85% vs 74%). Participants in the present study highlighted the benefits of receiving counseling on pain management, particularly in contrast to their past abortion care experiences, and the effect counseling had on their overall perceptions of quality of care, including reduced stress and pain during the abortion. Knowing they had the option to take additional pain medications as needed during the abortion process was reassuring for some participants. This is supported by literature indicating that pain expectations can shape the experience of pain [34]. Quality counseling regarding expectations for MA, access to health professionals for support during the process, and information about how and when to seek emergency care could help reduce anxiety and increase preparedness to manage the MA process.

The strengths of the present analysis include the inclusion of participants with a range of pain experiences with MA and from a diverse set of country settings where this topic has not yet been explored in depth. Little is known about women’s experiences with pain management in MA in general, but this is particularly the case outside of Europe and North America. Our study has some limitations. The small sample size, as well as the fact that we did not include patients younger than age 18 or those obtaining abortion care outside of facilities providing legal abortion services, limit the generalizability of our findings. Differences in pain by treatment group are explored in analyses of quantitative data from this study, and are therefore not the main purpose of this analysis. Findings from the qualitative data in this study were based on researcher and interviewer interpretations, and do not reflect participant interpretations beyond the transcripts. Some of the interview questions related to women’s autonomy and emotional state were not well understood by participants, leading to inconclusive findings for those domains. In addition, it is possible that social desirability bias [35] influenced the responses of some participants, perhaps leading them to underreport the severity of their pain or their use of nonpharmacological interventions, particularly if they viewed the interviewer as being associated with the clinical service.

## Conclusions

Combination medical abortion accounts for roughly 60–90% of all induced abortions in most of Europe, and misoprostol-only abortions are increasingly common in countries with restrictive laws on abortion [36]. Women consistently report pain as the worst aspect of MA. As an increasing proportion of women around the world rely on MA for their abortions, it is essential to improve their experiences with pain during the process in order to ensure quality of care. Incorporating a discussion about pain expectations and pain management strategies into pre-MA counseling and enabling access to information and support during the MA process could improve the quality of care experiences of patients. Findings from this study could assist in informing implementation efforts around pain management guidance for MA.

## Additional file

**Additional file 1.** Interview guide.

## Data Availability

The datasets generated and analyzed during the current study are not publicly available because they are qualitative in nature, include potentially identifiable information, and we wish to maintain the confidentiality of our participants, who were seeking a sensitive medical service. Please contact the corresponding author with any requests.

## Abbreviations

Ibu/met: Ibuprofen plus metoclopramide
MA: Medical abortion
NSAIDs: non-steroidal inflammatory drugs
RCOG: Royal College of Obstetrics and Gynecology
WHO: World Health Organization
y: Years (of age)
