# Supplementary material for: Experiences with pain of early medical abortion: qualitative results from Nepal, South Africa, and Vietnam
Source: BMC Womens Health. 2019 Oct 15;19:118. doi: 10.1186/s12905-019-0816-0 (PMC6794877; doi:10.1186/s12905-019-0816-0)
Supplement: Supplementary file 1 — Additional file 1. Interview guide. [file 12905_2019_816_MOESM1_ESM.docx]

***ENGLISH VERSION***

| A randomized, placebo-controlled study of two prophylactic medication approaches in addition to a pain control regimen for early medical abortion with mifepristone and misoprostol  **IN-DEPTH INTERVIEW** | | |
| --- | --- | --- |
| Facility Name ________________________________  City ______________________________________ | Interviewer’s Name____________________________  Interviewer’s ID: | |
| Today’s Date  Day Month Year | Time Interview Started  Hour Minutes | Time Interview Ended  Hour Minutes |
| Thank you for participating in this study. This interview will take about 45 minutes to 1½ hours. We have developed some questions to help guide our conversation. I will be asking about your experience with pain in general and about your recent abortion. Please keep in mind that there are no right or wrong answers to these questions. You are free to decline to answer any question(s), or stop the interview at any time. Our hope, in the end, is to interview different women with a range of experiences and thoughts on this subject. I want to stress again that you should not use anyone’s name during our discussion. We want all of the information you give us to remain confidential. Your opinions are so important to understand what women’s experiences are like obtaining an abortion and will help us find ways to improve services here. Do you have any questions before we begin the interview?  **[IF WOMAN CONSENTED TO RECORD INTERVIEW, SAY]:** Ok, I am going to begin recording the interview now. **[SAY THE STUDY ID AND DATE AT THE START OF THE RECORDING]** | | |

**General pain tolerance**

I’m going to start by asking about how you usually handle pain.

1. How would you describe your pain tolerance or ability to handle pain? Do you think you can tolerate more or less pain than most people?
2. I’d like you to think about a recent experience that caused you physical pain (other than your recent abortion). What makes your pain worse? [probe about having to do work or childcare, emotional distress, feeling sad or anxious]
3. What makes your pain better? Do you ever use pain medication? If so, what medication do you usually use? What level of pain do you usually reach before you take medication for your pain (Please show 0-10 scale)? What else do you do to cope with pain? [probe about massage, warm bath, walking, sleeping]
4. If you take pain medication, does it generally help your pain?

**Menstrual and pregnancy history**

1. Now I’d like to ask you about your menstrual period. Do you usually have painful menstrual periods? How bad is the pain? What do you do to cope with the pain? Do you take any medicine? Do you use massage or heat? If so, does it help?
2. Now I would like to ask you some questions about pregnancy. Have you been pregnant before this most recent pregnancy? How many times? Tell me about the number of times you have given birth? Have you had any spontaneous miscarriages? Have you had another abortion in addition to your most recent abortion?

Births ______ Miscarriages ______ Abortions ______

1. Can you tell me more about these events in your life?

In what order did they occur?

**[IF WOMAN HAS GIVEN BIRTH, ASK]:**

What was your experience with the pain of childbirth? What did you do cope with the pain? Did you take any medicine? If so, did it help?

**[IF WOMAN HAS HAD A PREVIOUS ABORTION OR MISCARRIAGE, ASK]:**

What was your experience with the pain of your previous [abortion/miscarriage]? What did you do to cope with the pain? Did you take any medicine? If so, did it help?

Now I’d like to focus on your recent abortion.

1. Please tell me about how you made the decision to have the abortion. Did anyone (else) support you in your decision to have an abortion? If so, who?

What about the man with whom you got pregnant… How was he involved?

1. Can you describe your experience during the abortion?

How did you feel physically? What was the pain like? In addition to the medicine you were given as part of the study, what did you do to manage the pain? [probe about massage, taking warm bath, etc.]

Where were you? Who, if anyone, was there with you? Did they know about the situation? How did they help you?

How did you feel emotionally? How did these emotional feelings affect your physical feelings, if at all?

1. How was your experience with the clinic staff where you had the abortion? Is there anything they could have done to improve your experience?
2. Looking back on your abortion experience now, how satisfied are you with the pain control you received? How did it compare to your menstrual cramps?

**[IF SHE HAD A PREVIOUS ABORTION, ASK]** How did it compare to your previous abortion?

**[IF SHE HAS HAD A DELIVERY, ASK]** How did it compare to your last delivery?

Is there anything you would have done differently to improve your experience with the abortion?

1. How much did you feel that you were in control of your abortion experience? Is there anything that could have been done to make you feel like you were more in control of the experience?
2. Looking back on your abortion experience now, what advice about pain control would you give a friend who had to have an abortion?

**Concluding questions**

We are now at the end of the interview. Just a few more questions….

1. Is there anything else about pain control for the abortion procedure that we haven’t talked about that you would like to discuss?
2. What was it like for you to talk to me about your experience?
3. Do you have any final questions for me about the interview?

Thank you again for agreeing to talk with me. Your opinions are so important to understanding what women’s experiences are like obtaining an abortion and will help us to improve services here.

**GIVE PARTICIPANT REIMBURSEMENT**

Interviewer notes or observations:

Interviewer notes or observations:
